# Limited significance of family history for presence of *BRCA1* gene mutation in Polish breast and ovarian cancer cases

**DOI:** 10.1007/s10689-012-9519-5

**Published:** 2012-03-01

**Authors:** Izabela Brozek, Magdalena Ratajska, Magdalena Piatkowska, Anna Kluska, Aneta Balabas, Michalina Dabrowska, Dorota Nowakowska, Anna Niwinska, Jadwiga Rachtan, Jan Steffen, Janusz Limon

**Affiliations:** 1 Department of Biology and Genetics, Medical University of Gdansk, Debinki 1, 80-211 Gdansk, Poland; 2 Department of Immunology, Maria Sklodowska-Curie Memorial Cancer Centre and Institute of Oncology, Warsaw, Poland; 3 Epidemiology Unit, Centre of Oncology, Maria Sklodowska-Curie Memorial Institute, Krakow, Poland

**Keywords:** BRCA1 mutation, Family history, Hereditary breast cancer, Hereditary ovarian cancer

## Abstract

It is estimated that about 5–10% of ovarian and 2–5% of all breast cancer patients are carriers of a germline *BRCA1* or *BRCA2* gene mutation. Most families with detected *BRCA1* or *BRCA2* gene mutation are qualified for molecular testing on the basis of family history of breast or ovarian cancers. The purpose of our study was to establish the frequency of positive family history of cancer in a series of Polish consecutive breast and ovarian cancer patients in two groups, with and without the *BRCA1* gene mutations. We analysed the prevalence of four of the most common *BRCA1* mutations: 5382insC (c.5266dupC), 300T>G (p.181T>G), 185delAG (c.68_69delAG) and 3819del5 (c.3700_3704del5). The patient group consisted of 1,845 consecutive female breast and 363 ovarian cancer cases. 19 out of 37 (51%) of *BRCA1*-positive ovarian cancer patients and 21 out of 55 (39%) *BRCA1*-positive breast cancer had negative family history of breast and/or ovarian cancer among first- and second-degree relatives. In ovarian cancer patients, negative family history was more frequent in those with 300T>G *BRCA1* gene mutation than in 5382insC carriers. This finding indicates the necessity of searching for 300T>G mutation in families with a single diagnosis of ovarian cancer in family. The high frequency of mutations detected in breast cancer patients lacking obvious family history shows that breast cancer patients should be qualified for genetic testing on the basis of wide clinical and pathological criteria.

## Introduction

It is currently accepted that about 5–10% of ovarian cancers and 2–5% of breast cancers have a hereditary background arising in *BRCA1/2* genes germline mutation carriers. Because of high costs and cumbersome procedure the mutation testing is offered to patients in whom family history indicates high probability of finding the mutation. Families with the increased risk of carrying a mutation can be identified by the large number of breast and ovarian cancer cases occurring among family relatives. However, some families with detected mutation have negative family history or limited family structure [1–4]. The purpose of our study was to establish the frequency and spectrum of *BRCA1* gene mutations in Polish consecutive breast and ovarian cancer patients in two groups: with positive and negative family history of *BRCA1*-related cancers. Our choice of the four *BRCA1* mutations: 5382insC (c.5266dupC), 300T>G (p.181T>G), 185delAG (c.68_69delAG), and 3819del5 (c.3700_3704del5) analysed in the present study reflects their high prevalence among Polish breast and/or ovarian cancer families and is based on the results of previous research [5]. As a strong founder effect is noted in Poland, these four *BRCA1* mutations comprise 70–90% of all *BRCA1* pathogenic alterations [6–12].

## Subjects and methods

Consecutive, newly-diagnosed female breast cancer cases were collected without regard to age or family history of breast and ovarian cancer in two provinces, Malopolska and Mazowsze, between 2003 and 2005. Consecutive invasive epithelial ovarian cancer cases were collected independently of age and family history in Pomorze and Malopolska between 1995 and 2005. The patients enrolled in the study were invited to provide blood samples and to complete a questionnaire including information on breast and ovarian cancers in first- and second-degree relatives regardless of their age. Family history was regarded as positive when, apart from the patient, at least one first- or second-degree relative suffered from breast or ovarian cancer regardless of their age. Analysis of the frequency of 5382insC (c.5266dupC), 300T>G (p.181T>G), 185delAG (c.68_69delAG), and 3819del5 (c.3700_3704del5) mutations of *BRCA1* gene was performed on groups of 1,845 breast and 363 ovarian cancer patients. The study received the approval of the local ethics committee review boards.

DNA was extracted from peripheral blood lymphocytes in accordance with standard procedure. Mutation analyses were done using a combination of different screening methods (restriction fragment length polymorphism—RFLP, single-strand conformation polymorphism—SSCP, denaturing high performance liquid chromatography—DHPLC, allele-specific PCR-ASA-PCR) followed by bidirectional sequencing of samples with abnormal patterns.

Comparisons between groups for statistical significance were performed by the use of χ^2^ and Fisher’s exact tests, as appropriate. P values less than 0.05 were considered statistically significant.

## Results

In the group of 1,845 unselected breast cancers collected in two Polish provinces, Malopolska and Mazowsze, the most frequent mutation was 5382insC (35/1845, 1.9%) followed by 300T>G (18/1486, 1.2%). We observed high frequency of negative family history in the group of carriers. In 21 out of 55 (38%) *BRCA1*-positive breast cancer cases no case of breast and/or ovarian cancer was observed in first- or second-degree relatives (Table 1).

**Table 1:** *BRCA1* gene mutations in consecutive breast cancer cases unselected for age and family history of breast and/or ovarian cancer and in those selected with negative family history from two Polish provinces

	Number of breast cancer cases with *BRCA1* mutation (frequency %)			
	Malopolska		Mazowsze	
	Unselected	Selected*	Unselected	Selected*
185delAG	0/755	0/541	2/705 (0.28)	0/453
300T>G	9/787 (1.14)	3/573 (0.52)	9/699 (1.29)	5/449 (1.11)
3819del5	0/554	0/341	0/688	0/448
5382insC	9/787 (1.14)	4/573 (0.70)	26/1058 (2.46)	9/775 (1.16)
Cumulative frequency of four mutations	1/44 (2.3)	1/182 (0.55)	1/25 (4.3)	1/44 (2.27)

* With negative family history for breast and/or ovarian cancer in I° or II° degree relatives

In the group of 363 unselected ovarian cancers analysed in two provinces, Malopolska and Pomorze, the most prevalent molecular change was 5382insC mutation (17/363, 4.7%), followed by 300T>G (12/363, 3.3%). 19 out of 37 (51%) of *BRCA1*-positive patients from these provinces had negative family history of breast and/or ovarian cancer among first- and second-degree relatives (Table 2). We also observed the difference in frequency of positive family history in *BRCA1*-positive patient groups depending on the type of the mutation. Negative family history was more frequent in ovarian cancer patients with 300T>G missense *BRCA1* mutation than in truncating 5382insC mutation carriers (*p* = 0.0025). Ten out of 12 *BRCA1* 300T>G-carriers had no first- or second-degree relative with breast and/or ovarian cancers whereas in the group of 17 *BRCA1* 5382insC-carriers only four had negative family history.

**Table 2:** *BRCA1* gene mutations in consecutive ovarian cancer cases unselected for age and family history of breast and/or ovarian cancer and with negative family history from two Polish provinces

	Number of ovarian cancer cases with *BRCA1* mutation (frequency %)			
	Malopolska		Pomorze	
	Unselected	Selected*	Unselected	Selected*
185delAG	2/205 (0.97)	2/162 (1.23)	1/151 (0.66)	0/96
300T>G	11/212 (5.19)	9/169 (5.33)	1/151 (0.66)	1/96 (1.04)
3819del5	1/160 (0.63)	1/117 (0.85)	4/151 (2.65)	2/96 (2.08)
5382insC	11/212 (5.19)	3/169 (7.78)	6/151 (3.98)	1/96 (1.04)
Cumulative frequency of four mutations	1/8 (12.5)	1/11 (9.09)	1/13 (7.69)	1/24 (4.17)

* With negative family history for breast and/or ovarian cancer in I° or II° degree relatives

## Discussion

Many centres undertook the screening programme to estimate the prevalence of *BRCA1* mutations in breast cancer cases unselected for age and family history of cancer. These investigations allowed us to evaluate the frequency of *BRCA1/2*-positive breast cancer cases at 2–5% [2, 13–18]. As this frequency is not sufficient to offer molecular testing to each breast cancer patient, in most genetic centres qualification for genetic testing is based on family history of cancer. In our study, almost 40% of *BRCA1*-positive breast cancers could be classified according to family history as not-familial. The great fraction of *BRCA1*-positive breast cancers without family history described in our study confirms previous observations reported in Polish and other populations [2, 6, 18]. The computer probabilistic models based on family history of breast and ovarian cancers are a useful tool for calculating *BRCA1/2* mutation probabilities but, for some small families with the mutation, will assign insufficient mutation probability. Misassignment of carriers to a test group can be a consequence of incomplete family information regarding ancestry, small family size or transmission through males. The previous data indicate inaccuracy in carrier prediction using computer models for families with a single breast cancer diagnosis [1, 19]. The high frequency of mutations detected in probants lacking obvious family history indicates the necessity to provide wide criteria for qualifying breast cancer women for genetic testing including pathological (high grade, medullary histology, negative oestrogen and progesterone receptors, and HER-2/neu status) and clinical (age, mammography) parameters of patients [20–22].

In this study, we revealed that, in Polish population, 51% of *BRCA1*-positive patients with ovarian cancer had negative family history of breast and/or ovarian cancer among I° and II° degree relatives. In the study provided by Risch et al. [3] the rate of *BRCA1*-positive ovarian cancers with negative family history was lower (19/75, 25%). This discrepancy can be explained by different familial criteria and spectrum of mutation in these two studies. As the *BRCA1* mutation can be detected in 10–15% of unselected ovarian cancer cases, positive family history is not necessary to qualify the patient for genetic tests [3, 23]. This observation indicates that, even in ovarian cancer cases with unknown or limited family structure, genetic tests should be offered. We observed that in Polish population negative family history is more frequent in the group of ovarian cancer patients with 300T>G *BRCA1* mutation than in 5382insC carriers. This finding indicates the necessity to search 300T>G mutation in families with a single diagnosis of ovarian cancer in family member including first- and second-relatives.

## Abbreviations

BRCA1: Breast cancer 1 gene
BRCA2: Breast cancer 2 gene
